# Impact of Smoking as a Risk Factor for Surgical Site Infections After Open Reduction and Internal Fixation (ORIF) of Distal Radius Fractures

**DOI:** 10.7759/cureus.94649

**Published:** 2025-10-15

**Authors:** Tariq Ahmad, Muhammad Arsalan Azmat Swati, Kamran Khan, Khalid Khan, Rahim Khan, Ziyad Ahmad, Shakir Ullah, Atizaz Ali Jan

**Affiliations:** 1 Department of Orthopaedic Surgery, Mardan Medical Complex, Mardan, PAK; 2 Department of Orthopaedics, Mardan Medical Complex, Mardan, PAK; 3 Department of Orthopaedics and Traumatology, Mardan Medical Complex, Mardan, PAK; 4 Department of Orthopaedics, Gajju Khan Medical College, Bacha Khan Medical Complex, Swabi, PAK; 5 Department of Trauma and Orthopaedics, University Hospital Crosshouse, Kilmarnock, GBR; 6 Department of Orthopaedics, Swat Group of Teaching Hospitals, Swat, PAK

**Keywords:** distal radius fracture, orif, postoperative infection, risk factors, smoking

## Abstract

Background: Smoking is a recognized risk factor for postoperative infections, but its specific impact on infection rates following distal radius fracture fixation remains underexplored.

Objective: To compare the postoperative infection rates following open reduction and internal fixation (ORIF) of distal radius fractures in smokers versus non-smokers.

Methodology: This retrospective cohort study was conducted at the Mardan Medical Complex, Mardan, Pakistan, over a three-year period (August 2020-July 2023). A total of 640 patients aged ≥18 years who underwent ORIF for distal radius fractures and had documented smoking status and 1-year postoperative follow-up were included. Patients were divided into smokers (n = 310) and non-smokers (n = 330). Data on demographics, comorbidities, surgical details, and microbiologically confirmed postoperative infections were analyzed. Chi-square test and multivariate logistic regression were used for statistical analysis in the IBM SPSS Statistics version 26.

Results: Infection rates were significantly higher among smokers (39 patients; 12.58%) compared to non-smokers (10 patients; 3.03%) (p < 0.001). Early infections (<30 days) occurred in 20 smokers (6.45%) and six non-smokers (1.82%), while late infections (≥30 days) were reported in 19 smokers (6.13%) and 4 non-smokers (1.21%). Multivariate logistic regression confirmed smoking as an independent predictor of postoperative infection, with smokers having more than a threefold increased risk compared to non-smokers (adjusted OR = 3.52, 95% CI: 1.68-7.37, p < 0.001). Poor glycemic control (HbA1c ≥7) also doubled the risk of infection (adjusted OR = 2.14, 95% CI: 1.01-4.55, p = 0.046). Additionally, prolonged operative time (>90 minutes) was associated with approximately a twofold increased risk (adjusted OR = 2.02, 95% CI: 1.03-3.97, p = 0.040).

Conclusion: Smoking significantly increases the risk of postoperative infections following ORIF of distal radius fractures.

## Introduction

One of the most frequent orthopedic injuries, especially in young people and the elderly after high-energy trauma, is a distal radius fracture [1]. Since it provides better anatomical alignment, quicker rehabilitation, and better functional results than conservative approaches, open reduction and internal fixation (ORIF) has become the standard therapy for unstable or displaced distal radius fractures [2,3]. However, ORIF introduces the risk of postoperative consequences, much like any surgical surgery, with infection being a key worry since it may delay recovery, impede function, and raise healthcare expenses [4].

Smoking is one of the well-known variables affecting the risk of postoperative infection [5]. Delays in bone healing, higher incidence of wound complications, and weakened immunological responses have all been repeatedly associated with cigarette smoking [6]. A less-than-ideal environment for wound healing and infection management is produced by nicotine and other toxic chemicals in tobacco, which can decrease peripheral blood flow, hinder tissue oxygenation, and change inflammatory responses [7]. The precise effect of smoking on infection rates after ORIF of distal radius fractures is still poorly understood in many populations, despite a well-established association between smoking and surgical site infections in a variety of orthopedic operations [8].

Furthermore, the distal radius may be particularly susceptible to postoperative complications because of its relatively superficial anatomical location, limited soft tissue coverage, and frequent exposure to direct trauma [9]. These factors make wound healing more challenging in general, and when combined with the detrimental vascular and immunologic effects of smoking, the risk of surgical site infections may be further amplified. Therefore, evaluating smoking as a potential modifier of infection risk in distal radius ORIF is clinically relevant and warrants focused investigation [10,11].

The infection rates after ORIF of distal radius fractures in smokers and non-smokers are compared in this retrospective cohort research. The project intends to provide information that may guide clinical judgments, enhance patient counseling, and support customized postoperative care plans by analyzing an actual clinical dataset from orthopedic clinics. This study compared the postoperative infection rates following ORIF of distal radius fractures in smokers versus non-smokers through retrospective analysis of patient records.

## Materials and methods

Study design and setting

This retrospective cohort study was conducted at the Mardan Medical Complex, a tertiary care teaching hospital in Mardan, Pakistan. The study spanned three years from August 2020 to July 2023. The objective was to compare postoperative infection rates following ORIF of distal radius fractures in smokers versus non-smokers, utilizing existing patient records from the hospital database.

Inclusion and exclusion criteria

Patients were eligible for inclusion if they were aged ≥18 years, had undergone ORIF for distal radius fractures during the study period (August 2020-July 2023), and had complete medical records documenting smoking status at the time of admission as well as at least 12 months of postoperative follow-up. Only cases with microbiologically confirmed documentation of surgical site infections were included. Patients were excluded if they had open fractures requiring complex reconstruction or polytrauma necessitating multiple concurrent surgeries, were immunocompromised (e.g., receiving long-term corticosteroids, chemotherapy, or living with HIV), or had pre-existing surgical site infections. Records with incomplete data regarding smoking status, follow-up, or infection outcomes were also excluded.

Sample size

An initial total of 748 patient records was reviewed for potential inclusion in the study. However, 108 cases were excluded due to incomplete follow-up records, which were often the result of inadequate postoperative documentation, missing infection outcomes, or records indicating that patients did not attend their scheduled follow-up appointments. Consequently, the final analysis contained 640 patient records with full and eligible data, including a one-year follow-up. Of these, 330 patients were categorized as non-smokers based on their medical records' reported smoking status, whereas 310 patients were identified as current smokers at the time of admission. The comparison of postoperative infection rates was based on these two cohorts.

Due to the retrospective, single-center design and the intention to include all consecutive patients who met the inclusion criteria during the three-year study period (August 2020 to July 2023), convenience sampling was used. As the study was exploratory and aimed to capture real-world clinical outcomes in a typical orthopedic setting, no formal a priori power or sample size calculation was conducted. Nevertheless, the final sample size is comparable to those used in previous retrospective observational studies examining infection rates after orthopedic surgeries [12].

Additionally, a post-hoc power analysis was performed to assess the adequacy of the study sample. Based on the observed difference in infection rates between smokers (12.58%) and non-smokers (3.03%), with group sizes of 310 and 330, respectively, the study had an achieved statistical power of approximately 99.5% (α = 0.05, two-sided). For reference, a minimum of about 120 patients per group (≈240 total) would have been required to detect this difference with 80% power. Therefore, the final sample size substantially exceeded this threshold, supporting the robustness of the analysis.

Data collection

Hospital computerized medical records and surgery logs were the sources of the data. Patient demographics, such as age and gender, smoking status (classified as current smoker or non-smoker), and comorbidities, such as diabetes mellitus and hypertension, were among the data gathered. The type of distal radius fracture (whether closed or open, and if intra-articular components were involved), surgical details (such as the fixation method and duration of surgery), and postoperative infections were also recorded.

To determine infection status, only cases fulfilling the Centers for Disease Control and Prevention (CDC) criteria [13] for surgical site infection were included. This required both positive microbiological culture reports (including organism identification and antibiotic susceptibility profiles) and supporting clinical evidence such as erythema, swelling, purulent discharge, localized pain, or systemic signs of infection. This approach was taken to minimize the risk of misclassifying colonization as infection.

To further reduce the influence of potential confounders, data on nutritional status markers (e.g., serum albumin levels or clinician-documented malnutrition) and perioperative glycemic control (e.g., HbA1c values for diabetic patients) were gathered when available. Follow-up data for all included patients were reviewed for a maximum of one year after surgery, in order to capture both early and late-onset postoperative infections, particularly those associated with internal fixation hardware. Although this extended follow-up improves the detection of delayed infections, it also carries the limitation of potential attrition and variation in record completeness. All patient data were anonymized prior to analysis to maintain confidentiality and comply with ethical research guidelines.

Statistical analysis

IBM SPSS Statistics version 26 (IBM Corp., Armonk, NY) was used for the statistical analysis. Frequencies and percentages were used to describe categorical data, such as smoking status and infection outcomes, and the chi-square test was used to compare groups. Depending on the data distribution, independent t-tests were used to evaluate continuous variables, including age, which were reported as mean ± standard deviation. For all inferential statistics, a significance criterion of p < 0.05 was established. Multivariate logistic regression analysis was used to find independent predictors of postoperative infection while controlling for relevant confounders such as age, comorbidities, fracture type, glycemic management, and nutritional condition.

Ethical approval

This study was approved by the Institutional Review Board (IRB) of the Mardan Medical Complex, Mardan (No. 321/ORT/BKMC, dated 14.07.2020). As the study involved retrospective analysis of anonymized medical records, the requirement for informed consent was waived by the ethics committee.

## Results

The mean age of smokers (n = 310) of the 640 patients included was 44.8 years, whereas that of non-smokers (n = 330) was 40.1 years (Table 1). Male patients made up the majority of the smoking group (291 patients, 93.87%), whereas female patients made up the majority of the non-smokers (229 patients, 69.39%). A total of 59 smokers (19.03%) and 57 non-smokers (17.27%) had diabetes mellitus. Among the diabetic population, 26 smokers (44.07%) and 23 non-smokers (40.35%) had poor glycemic control (HbA1c ≥7%). A total of 61 non-smokers (18.48%) and 71 smokers (22.90%) had hypertension. Additionally, 42 smokers (13.55%) and 30 non-smokers (9.09%) had low blood albumin levels (<3.5 g/dL), whereas 47 smokers (15.16%) and 36 non-smokers (10.91%) had nutritional deficiencies. Compared to non-smokers (3.86 g/dL), smokers had a lower mean serum albumin (3.70 g/dL).

**Table 1:** Baseline Characteristics of Study Population by Smoking Status (N = 640). Data presented as mean ± SD or n (%). Independent samples t-test used for continuous variables, and Chi-square test for categorical variables.

Category	Variable	Smokers (n = 310)	Non-Smokers (n = 330)	p-value
Demographics	Age (mean ± SD, years)	44.8 ± 12.9	40.1 ± 14.2	0.006 (t = 2.75)
	Male (n, %)	291 (93.87)	101 (30.61)	<0.001 (χ² = 320.6)
	Female (n, %)	19 (6.13)	229 (69.39)	—
Metabolic Profile	Diabetes Mellitus (n, %)	59 (19.03)	57 (17.27)	0.540 (χ² = 0.37)
	Mean HbA1c (% in diabetics, mean ± SD)	7.6 ± 1.2	7.2 ± 1.0	0.030 (t = 2.20)
	Poor Glycemic Control (HbA1c ≥7, n, %)	26/59 (44.07)	23/57 (40.35)	0.710 (χ² = 0.14)
	Hypertension (n, %)	71 (22.90)	61 (18.48)	0.190 (χ² = 1.69)
Nutritional Status	Nutritional Deficiency (n, %)	47 (15.16)	36 (10.91)	0.100 (χ² = 2.71)
	Low Serum Albumin <3.5 g/dL (n, %)	42 (13.55)	30 (9.09)	0.080 (χ² = 3.06)
	Mean Serum Albumin (g/dL, mean ± SD)	3.70 ± 0.47	3.86 ± 0.35	0.010 (t = -2.58)

There were 272 smokers (87.74%) and 300 non-smokers (90.91%) who had closed fractures, and 38 smokers (12.26%) and 30 non-smokers (9.09%) who had open fractures (Gustilo Type I), shown in Table 2. A total of 136 non-smokers (41.21%) and 145 smokers (46.77%) had intra-articular fracture involvement. Additionally, 260 smokers (83.87%) and 277 non-smokers (83.94%) had volar plating, whereas 50 smokers (16.13%) and 53 non-smokers (16.06%) had dorsal plating. For smokers, the average operation time was 80.2 minutes, whereas for non-smokers, it was 77.1 minutes.

**Table 2:** Fracture and Operative Characteristics by Smoking Status. *Only Gustilo Type I fractures included. Data presented as mean ± SD or n (%). Independent samples t-test used for continuous variables, and Chi-square test for categorical variables.

Category	Variable	Smokers (n = 310)	Non-Smokers (n = 330)	p-value
Fracture Type	Closed Fracture (n, %)	272 (87.74)	300 (90.91)	0.220 (χ² = 1.50)
	Open Fracture (Type I only, n, %)*	38 (12.26)	30 (9.09)	—
Involvement	Intra-articular Involvement (n, %)	145 (46.77)	136 (41.21)	0.180 (χ² = 1.80)
Fixation Type	Volar Plate (n, %)	260 (83.87)	277 (83.94)	0.980 (χ² = 0.001)
	Dorsal Plate (n, %)	50 (16.13)	53 (16.06)	—
Operative Details	Duration of Surgery (minutes, mean ± SD)	80.2 ± 19.7	77.1 ± 22.8	0.100 (t = 1.64)

Ten non-smokers (3.03%) and 39 smokers (12.58%) had postoperative infections (Table 3). Twenty smokers (6.45%) and six non-smokers (1.82%) had early infections (within 30 days), while 19 smokers (6.13%) and four non-smokers (1.21%) had late infections (≥30 days). *Staphylococcus aureus* (*S. aureus*) was the most often isolated pathogen, occurring in 15 smokers and 6 non-smokers. Methicillin-resistant* S. aureus* (MRSA) was found in one non-smoker and eight smokers. Six smokers and one non-smoker had *E. coli *isolated from them. Five smokers and none of the non-smokers had *Pseudomonas aeruginosa*. Two non-smokers and five smokers had polymicrobial infections.

**Table 3:** Postoperative Infection Rates and Microbiological Profiles by Smoking Status. Data presented as n (%). Chi-square test applied to categorical variables. MRSA: methicillin-resistant *Staphylococcus aureus*

Category	Variable	Smokers (n = 310)	Non-Smokers (n = 330)	p-value
Infection Rates	Total infections (n, %)	39 (12.58)	10 (3.03)	<0.001 (χ² = 18.40)
	Early infection <30 days (n, %)	20 (6.45)	6 (1.82)	0.002 (χ² = 9.50)
	Late infection ≥30 days (n, %)	19 (6.13)	4 (1.21)	<0.001 (χ² = 13.10)
Common Isolated Pathogens	*Staphylococcus aureus* (n)	15	6	—
	MRSA (n)	8	1	—
	*E. coli* (n)	6	1	—
	*Pseudomonas aeruginosa* (n)	5	0	—
	Polymicrobial (n)	5	2	—

According to Table 4, smoking was substantially associated with postoperative infection, with an adjusted odds ratio (OR) of 3.52 (95% CI: 1.68-7.37, p < 0.001), indicating that smokers were more than three times as likely to develop an infection compared to non-smokers. Poor glycemic control (HbA1c ≥7%) was also an independent predictor of infection (OR = 2.14, 95% CI: 1.01-4.55, p = 0.046), suggesting more than double the risk relative to patients with adequate control. Surgeries lasting longer than 90 minutes were associated with approximately a twofold increase in infection risk (OR = 2.02, 95% CI: 1.03-3.97, p = 0.040). While open fractures, diabetes, hypertension, and nutritional deficiencies were associated with higher odds, these associations did not reach statistical significance.

**Table 4:** Multivariate Logistic Regression for Predictors of Postoperative Infection. Multivariate logistic regression model applied. Data presented as adjusted odds ratio (OR) with 95% confidence interval (CI).

Variable	Adjusted OR	95% CI	p-value
Smoking (Yes vs. No)	3.52	1.68 – 7.37	<0.001
Diabetes mellitus	1.48	0.78 – 2.83	0.230
Poor glycemic control (HbA1c ≥7)	2.14	1.01 – 4.55	0.046
Hypertension	1.31	0.69 – 2.48	0.400
Nutritional deficiency	1.71	0.87 – 3.35	0.120
Open fracture	1.93	0.87 – 4.26	0.105
Duration of surgery >90 minutes	2.02	1.03 – 3.97	0.040

## Discussion

Our study demonstrates a clear association between smoking and postoperative infections after ORIF of distal radius fractures. Smokers experienced infection rates nearly four times higher than non-smokers, with both early and late infections occurring more frequently among tobacco users. This trend aligns with existing literature, which has consistently identified smoking as a substantial risk factor for surgical site infections in orthopaedic surgery. For example, Magalhães et al. reported that smokers undergoing orthopedic procedures had infection rates two to three times higher than their non-smoking counterparts [14]. More recent systematic reviews and meta-analyses have also confirmed smoking as one of the most consistent modifiable predictors of surgical site infections across orthopedic subspecialties [15]. The biological plausibility is well established, as nicotine impairs vascular perfusion, tissue oxygenation, leukocyte activity, and collagen synthesis, leading to delayed wound healing and increased infection susceptibility [6,7].

Microbiological analysis further strengthened these observations. *S. aureus* was the most common pathogen, with MRSA disproportionately found in smokers. This aligns with prior reports on orthopedic implant-related infections, which consistently highlight *S. aureus* as the leading causative organism [16-20]. The higher prevalence of MRSA in smokers may be explained by delayed wound healing, prolonged hospital stays, and recurrent antibiotic exposures-factors that increase the likelihood of resistant pathogen colonization [17]. These results underscore the importance of tailored perioperative antibiotic prophylaxis and vigilant infection monitoring in high-risk groups such as smokers.

In addition to smoking, other modifiable and procedural factors also influenced infection risk. Poor glycemic control and prolonged operating times were both associated with higher infection likelihood. These associations are well-supported by previous research linking perioperative hyperglycemia to impaired neutrophil function and reduced tissue oxygen delivery, which in turn increases the risk of surgical site infection [17,18,19]. Similarly, extended surgical duration has been repeatedly implicated as a risk factor due to prolonged exposure, increased tissue trauma, and a higher probability of intraoperative contamination [5,16].

Interestingly, although open fracture type and nutritional deficits showed trends toward higher infection risk, they did not reach statistical significance in our cohort. Comparable findings have been reported by He et al., who noted that hypoalbuminemia may signal an increased risk of wound complications, but is not always independently predictive once other covariates are considered [20]. Other studies, however, have reported stronger associations between malnutrition, immune suppression, and surgical site infection risk, suggesting that larger multicenter analyses are warranted to clarify this relationship [19].

The infection incidence among non-smokers in our study was consistent with that reported in clean orthopedic surgeries involving internal fixation [4,9]. However, the markedly higher infection burden in smokers highlights the clinical relevance of preoperative risk modification. Our findings strongly support the integration of structured smoking cessation programs into routine surgical care for trauma patients. Beyond reducing infection risk, such programs have also been shown to improve overall bone healing and long-term functional outcomes [6,17].

Strengths and limitations

This study has several notable strengths. The relatively large sample size (n = 640) and the near-equal distribution of smokers and non-smokers allowed for a robust comparison. The 12-month follow-up enhanced our ability to capture both early and late postoperative infections, particularly those associated with hardware-related complications. Furthermore, the use of multivariate analysis to account for key confounders - including diabetes, glycemic control, nutritional status, and surgical variables - strengthened the internal validity of our findings. By focusing on distal radius fractures, which present unique anatomical considerations such as relatively limited soft tissue coverage and variable vascular supply, this study provides additional insight into upper limb infection risk in comparison with other orthopedic sites.

This study has some limitations. The retrospective design introduces risks of incomplete documentation and misclassification of both smoking status and infection outcomes. While smoking status was recorded from medical records and patient history, data on smoking intensity (e.g., pack-years), perioperative cessation status, or nicotine replacement therapy were unavailable, which could have influenced outcomes. The use of single-center data and convenience sampling also restricts external generalizability, especially to populations with different smoking prevalence or perioperative care protocols. Moreover, although microbiological confirmation was performed, detailed grading of infection severity, treatment responses, and long-term functional outcomes was not systematically assessed, limiting the depth of clinical interpretation. Future prospective multicenter studies with standardized infection severity scoring, quantification of smoking exposure, and evaluation of cessation interventions would provide more definitive insights into these associations.

## Conclusions

This study demonstrates that smoking is a major risk factor for postoperative infections following distal radius fracture fixation, underscoring the importance of incorporating smoking cessation interventions into preoperative care. Poor glycemic control and prolonged operative time also emerged as modifiable contributors to infection risk, while nutritional status and fracture type showed weaker associations. These findings highlight the need for comprehensive perioperative management strategies to reduce infection risk, particularly in high-risk patient groups.
